# A case of polymyositis associated with papillary thyroid cancer: a case report

**DOI:** 10.1186/1757-1626-1-289

**Published:** 2008-10-30

**Authors:** Dimitrios Kalliabakos, Apostolos Pappas, Emmanuel Lagoudianakis, Artemisia Papadima, John Chrysikos, Christos Basagiannis, Maria Tsakoumagou, Yasemi Skanelli, Andreas Manouras

**Affiliations:** 1 401 General Military Hospital, Mesogeion Avenue 138, 115 25, Athens, Greece; 2 First Department of Propaedeutic Surgery, Hippocrateion Hospital, Athens Medical School, Q. Sophia 114, 11527, Athens, Greece; 3 General Surgery, Agamemnonos 17, Alimos, 17456, Athens, Greece

## Abstract

Differentiated thyroid cancer is rarely associated with paraneoplastic events. Polymyositis, an autoimmune inflammatory myopathy, can be manifested as a paraneoplastic syndrome (PS). We report a case of a young woman who developed progressive proximal muscle weakness one and a half year after a total thyroidectomy for papillary thyroid cancer. Clinical features, laboratory results and muscle biopsy led us to the diagnosis of polymyositis, possibly related to her previous malignancy. A search for recurrence of the thyroid carcinoma or other underlying malignancy was fruitless. The patient improved slowly but almost completely after about 6 months of immunosupressive therapy, which she is still receiving.

## Background

Polymyositis (PM), along with dermatomyositis (DM), are classified as idiopathic inflammatory myopathies [1]predominantly affecting adults. Patients usually report increasing difficulty with everyday tasks due to progressive and often symmetric muscle weakness. The diagnosis is based on a combination of clinical, laboratory and muscle biopsy findings [2]. Both disorders may be linked with malignancy, with DM patients facing a greater risk. We present a case of a young woman who developed PM one and a half year after thyroidectomy for a papillary thyroid carcinoma. To date only two cases of papillary thyroid cancer have been reported to be complicated with inflammatory myositis, specifically DM [3,4].

## Case report

A 31 – year – old woman was admitted to our hospital complaining of about 2-month duration myalgias of the shoulder and neck muscles and progressive difficulty combing her hair, climbing stairs, walking uphill and rising from a chair. She had no fever, arthralgia or arthritis, cough, dyspnea, rash, difficulty with swallowing, paresthesias, abdominal pain, or other symptoms. Her physical examination was remarkable for 3/5 muscle strength in the upper and lower extremities confined to the proximal muscle groups. The tendon reflexes, muscular tone and sensation were normal throughout and there were no skin lesions. Chvostek's and Trousseau's sign were also negative, as was the rest of the examination.

A prior diagnoses of Hashimoto thyroiditis (HT) was made several years ago for which the patient did not receive any medication. One and a half year ago the patient was diagnosed with differentiated thyroid cancer and treated with total thyroidectomy and regional neck dissection. On histology, the tumor was a 3.5 cm papillary cancer, showed a mainly follicular pattern of development, capsule and vascular invasion. One of the seven dissected regional nodes was also invaded by tumor cells. The patient received hormone suppression with 225 μg of levothyroxine daily with Thyroid Stimulating Hormone (TSH) values ranging from 0.01 – 1 mU/L and six months postoperatively, 100 mCi of ^131^I were administered for thyroid remnant ablation, after preparation with a two-dose regimen of recombinant human TSH. One year postoperatively, a whole body scan with 5 mCi radiodine was negative for residual or metastatic disease, while continuing the aforementioned suppressive dose of levothyroxine daily.

The patient did not smoke, drink alcohol or use illicit drugs and her family history was negative for neuromuscular diseases.

At the present admission, laboratory investigation showed a 40-fold elevation of plasma creatine kinase (CK) level, as well as about 5-fold elevation of aldolase, and 3-fold transaminase and lactic dehydrogenase (LDH) levels. A mild hypocalcaemia was found (7.8 mg/dl, normal 8.1–10.2) with normal serum albumin (48 g/l). Serum phosphorus was high-normal (4.5 mg/dl, normal 2.3–4.6), serum creatinine was 0.9 mg/dl, serum magnesium was low: 0.6 mmol/l (normal 0.7–1.1). Serum Parathyroid hormone was normal (23 pg/ml, normal 15–75) and 25(OH) vitamin D was normal (30 nmol/l, normal 25–75). Antinuclear antibodies were detected in low titer (1/80) with standard immunofluorescence, whereas the myositis-specific antibody anti-Jo-1 and the ENAs were negative. The anti-AchR antibodies were also negative. The TSH level was < 0.01 mU/L.

**Figure 1:**
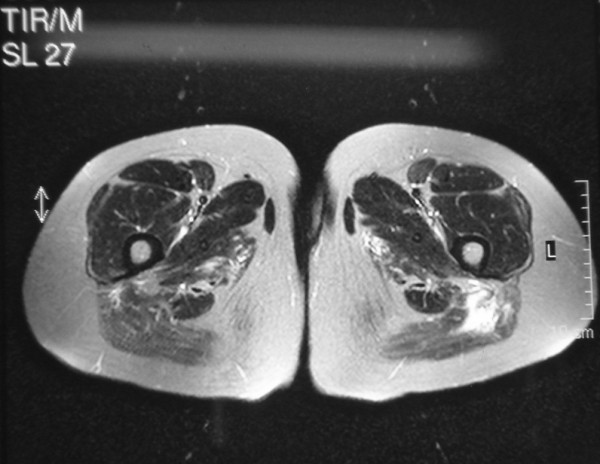
**Magnetic resonance imaging (MRI) of the gluteal and posterior femoral muscles revealed increased signal intensity on T1 sequencing, attributed to edema and inflammation.** Along with MRI, electromyography and muscle biopsy findings were compatible with the diagnosis of polymyositis

A Magnetic resonance imaging (MRI) of the gluteal and posterior femoral muscles was performed and revealed increased signal intensity on T1 sequencing, attributed to edema and/or inflammation [Figure 1].

The Electromyography (EMG) of upper and lower limbs showed myopathic changes to all the examined proximal muscles, e.g. short – duration, low-amplitude polyphasic potentials and positive sharp waves, more prominent at the pelvic girdle muscles.

Finally, we performed an open biopsy of her left deltoid muscle (which was not tested with EMG), which on light microscopy showed mild fiber atrophy and necrosis, degeneration and regeneration, as well as an inflammatory infiltrate composed mainly of CD4+ lymphocytes scattered in between the muscle fibers and within the fascicles. No evidence of vasculitis or other metabolic myopathy was detected and the dystrophin staining did not detect dystrophic changes. Assessment with immunofluorescence did not reveal any immune deposits at the skin or muscle.

The patient was started on prednisone 0.5 mg/kg/d and azathioprine 2 mg/kg/d along with calcium and vitamin D supplementation. With the aim of localizing persistent and/or recurring thyroid disease or a new underlying malignancy, the patient was evaluated with whole body radioiodine scan, neck ultrasound, chest X-ray, abdominal Computed tomography, transvaginal ultrasound and mammography, with no evidence of disease. Her serum thyroglobulin was < 0.1 ng/ml (normal range 0.1 – 1). She improved very slowly but steadily and after about 6 months of slow tapering the prednisone was stopped while continuing the same azathioprine dose. She had regained almost normal muscular strength, along with complete normalization of the muscle enzymes.

## Discussion

Both PM and DM, as defined clinically, have prevalence rates estimated at approximately one per 100,000 in the general population. There is a female to male predominance of about 2:1. The peak incidence in adults occurs between the ages of 40 and 50, but individuals of any age may be affected [5]. Histologic features of DM and PM include muscle fiber necrosis, degeneration and regeneration, and inflammatory cell infiltration. In PM cellular infiltrates are located predominantly within the fascicle, consisting of cytotoxic CD8+ T-cells and macrophages, whereas in DM, CD4+ T-cells, macrophages and B-cells are found mainly perifasicular and often perivascular[1]. Furthermore in DM intravascular depositions of membrane attack complex leads to perifascicular ischemic lesions and subsequent reduction in the capillary density [6,7]. The predominance of T CD4+ lymphocytes in the muscular biopsy of our patient is not in favour of myositis, however the rest histological, laboratory and clinical features appears to be highly supportive of the diagnosis [2]

The major clinical manifestations of these inflammatory myopathies are muscle weakness. In addition patients with DM have characteristic skin lesions. Muscle weakness is typically symmetric and predominantly proximal [8]. Elevations in serum CK, LDH, aldolase, and aminotransferases occur in most patients. In severe cases, the serum CK concentration may be elevated 50-fold. Although a correlation between the severity of the weakness and the height of elevation in serum muscle enzymes may be seen, the degree of muscle dysfunction may be much greater than the enzyme levels would suggest [9]. Autoantibodies are found in a majority of patients. Among the myositis-specific antibodies, anti-histidyl-tRNA synthase antibodies (eg, the anti-Jo-1 antibody) has been associated with interstitial lung disease, Raynaud phenomenon, fever, arthritis, and mechanic's hands, a syndrome known as the anti-synthetase syndrome [10]. EMG shows evidence of muscle irritability. EMG findings are helpful in confirming the presence of a myopathic process and in indicating which muscle groups are most involved. This information can be valuable in selecting a site for muscle biopsy. MRI may be useful if physical examination and EMG fail to identify a suitable target for muscle biopsy. MRI may also be useful in the longitudinal follow-up of patients, as an adjunct test to assess treatment responses and to diagnose disease flares [11].

The association between the idiopathic inflammatory myopathies and the development of malignancy has been appreciated for nearly a century, but its meaning and significance have remained unclear. Perhaps the most robust data describing the link between myositis and malignancy was published by Hill and colleagues [12] in 2001. The authors identified 618 dermatomyositis and 914 polymyositis patients. Cancer was detected in approximately 30% of DM and 15% of PM patients, with over 60% of tumors diagnosed after the diagnosis of myopathy. Both groups were noted to have increased cancer risks compared with the general population (standardized incidence ratio of 3.0 for DM, 1.4 for PM). The majority of cancers were diagnosed within one year of the development of myositis, and as in previous observational studies, the most common cancer types noted were adenocarcinomas, which accounted for 70% of all associated tumors in both DM and PM patients. Other studies have reported a frequency of malignancy of 15 to 25 percent [13].

The spectrum of malignancies associated with DM or PM parallels the distribution in the general population with the possible exceptions of an increased frequency of cervical, lung, ovarian, pancreatic, bladder, and gastric carcinomas, and with non-Hodgkin lymphoma [12-14].

Cancer can be diagnosed before, at the time of, or after the diagnosis of myositis with a peak incidence occurring within the two year period before and after the development of myositis [14]. Also, in some patients, the myositis is first diagnosed at a time of recurrence of a previously diagnosed cancer, while in others the myositis reactivates at a time when the cancer first becomes obvious [15].

Our patient was diagnosed with PM based on clinical presentation, elevated CK and muscle biopsy results. Thyroid cancer preceded the development of PM in our case, and this temporal relation suggests a PS, however due to the absence of paraneoplastic antibodies a firm diagnosis is difficult to be made. Nevertheless according to Graus F. et al [16], PM, a "nonclassical" paraneoplastic neurologic syndrome in that study, is possibly paraneoplastic, even if "well-characterized" paraneoplastic antibodies (anti-Hu, CV2, Ri, Ma2, and amphiphysin) are absent, provided that it occurs within two years of malignancy. Furthermore, since PSs are considered to be immune mediated perhaps the presence of a prior medical history of HT, a well-known autoimmune disorder [17], could act as a promoting factor indicating a deranged immunological background.

In conclusion, this is the first report of PM associated with differentiated thyroid cancer. Awareness of a potential PM complication is important because it can be confused with other causes of muscle weakness in the case of malignancy and/or after a thyroidectomy – such as myasthenic syndromes, hypothyroidism, hypocalcemic myopathy, etc.- and because prompt diagnosis of PM with early initiation of steroid and immunosuppressive therapy in such a clinical setting may avoid subsequent sequel.

## Abbreviations

TSH: Thyroid Stimulating Hormone; PT: Parathyroid hormone; MRI: Magnetic resonance imaging; EMG: Electromyography; CXR: Chest X-ray; DM: dermatomyositis; PM: polymyositis.

## Consent

Written informed consent was obtained from the patient for publication of this case report and accompanying images. A copy of the written consent is available for review by the Editor-in-Chief of this journal.

## Competing interests

The authors declare that they have no competing interests.

## Authors' contributions

All authors contributed equally to this work, DK and A Pappas analyzed and interpreted the patient data, EL and A Papadima performed the histological examination of the muscle biopsy, JC, CB and MT reviewed the current literature, YS and AM contributed in writing the manuscript. All authors read and approved the final manuscript.
